# A Case of Wellens' Syndrome

**DOI:** 10.7759/cureus.50736

**Published:** 2023-12-18

**Authors:** Jessica Kim, Shayan Zaheer, Hanad Omar

**Affiliations:** 1 Internal Medicine, Portsmouth Regional Hospital, Portsmouth, USA; 2 Internal Medicine, Aventura Hospital and Medical Center, Aventura, USA

**Keywords:** wellens' syndrome, left anterior descending, ekg, cardiology, acute coronary syndrome, wellens

## Abstract

This is a clinical case of a 43-year-old male with a past medical history notable for tobacco use disorder who presented to the emergency department for evaluation of typical chest pain. ECG was consistent with Wellens' syndrome with deeply inverted T waves in the anteroseptal leads. Coronary angiography confirmed a proximal left anterior descending coronary artery lesion, alongside other areas of coronary artery disease, and the patient was treated with surgical revascularization. There are specific ECG findings consistent with Wellens' syndrome that are important for all physicians to recognize, as they are associated with a serious cardiovascular condition that necessitates early invasive cardiac catheterization.

## Introduction

Wellens' syndrome is a type of acute coronary syndrome (ACS) that is associated with critical stenosis involving the left anterior descending coronary artery (LAD). Patients will oftentimes present with chest pain and classic ECG findings, including biphasic T waves in leads V2 and V3 (type A) or deep T wave inversions in leads V2 and V3 (type B), which can also extend to the other precordial leads. Other criteria include EKG findings with isoelectric or minimal ST-segment elevation (<1 mm), ST-segment depression, and EKG findings that exclude pathologic Q waves, left or right bundle branch blocks, left or right ventricular hypertrophy, and poor R-wave progression [1].

## Case presentation

**Figure 1:**
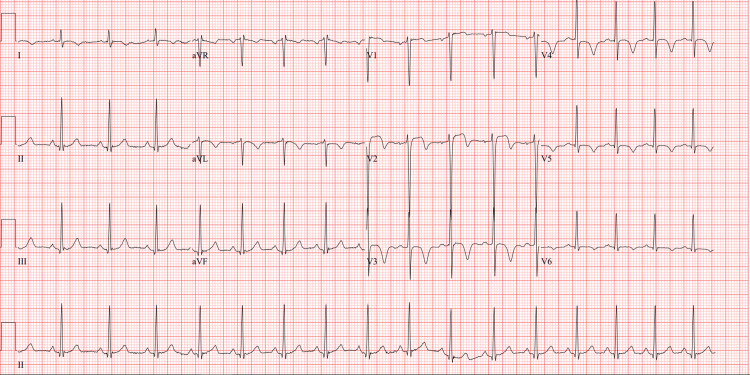
EKG showing biphasic T waves in V2 and deeply inverted T waves in V3-V5.

A 43-year-old male with a past medical history notable for tobacco use disorder presented to the emergency room for evaluation of chest pain. He had reported first experiencing chest pain eight days prior to admission while pulling a water heater out of a basement. His chest pain was described as substernal pain that radiates to his left jaw and arm and is associated with diaphoresis. The pain resolved after he sat down and drank some cold water. His next episode of chest pain occurred four days prior to admission, similar in nature to the previous episode while he was walking. After this second episode, he began to have daily chest pain that would occur with activity and improve with rest, so he presented to the emergency room. At the outside hospital, his initial high-sensitivity troponin was 78 ng/L and ECG was reported to show deep T waves in the anterior leads. He was loaded with aspirin and started on a heparin drip prior to being transferred to our hospital for further evaluation and management of ACS. ECG on arrival at our facility showed inverted T waves in the lateral leads and deeply inverted T waves in the anteroseptal leads, consistent with Wellens' syndrome (Figure 1).

**Figure 2:**
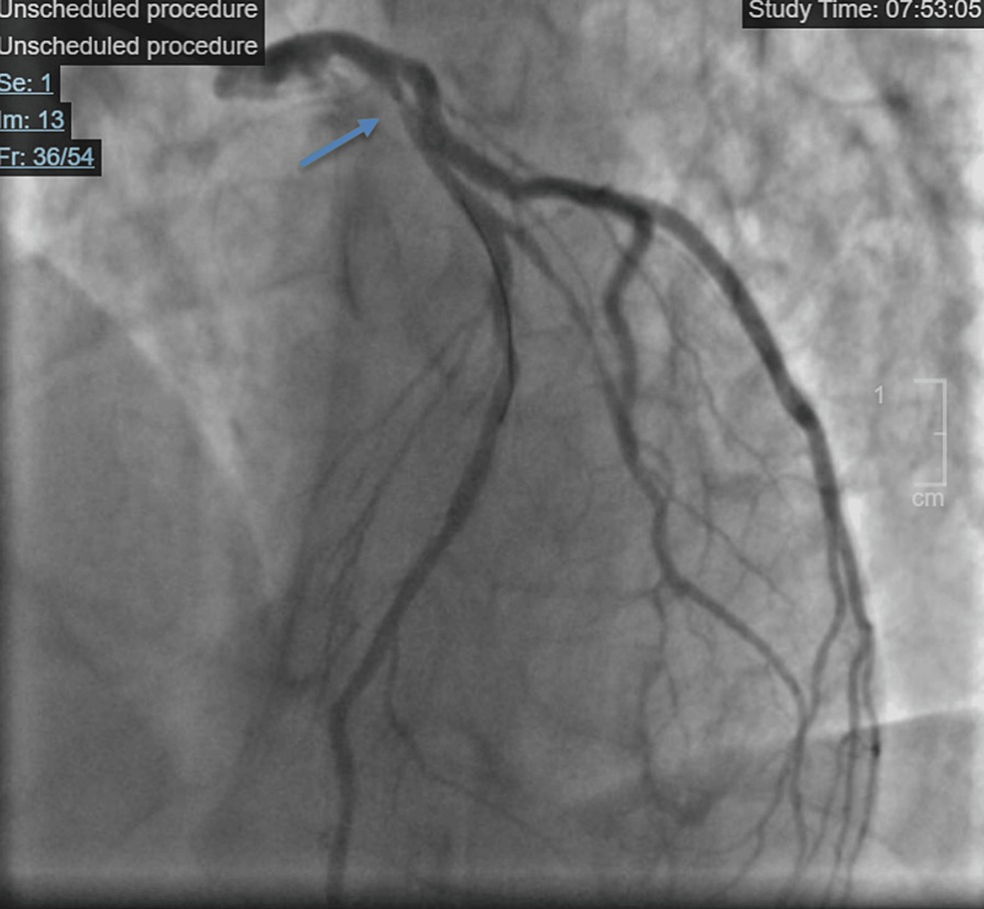
Arrow pointing to proximal LAD stenosis noted on coronary angiography. LAD: left anterior descending artery.

High-sensitivity troponin on arrival to our facility was 143.9 ng/L at 14:25, then 156.5 ng/L at 16:10, and 154.5 ng/L at 18:55 (normal troponin reference range < 78.5 ng/L) when checked serially. Transthoracic echocardiogram showed moderately reduced left ventricular systolic function with ejection fraction estimated at 35-40% and hypokinesis of the mid-apical anterior, mid anteroseptal, apical septal, apical lateral, and apex walls. There was no evidence of significant valvular pathologies, more specifically, no noted aortic stenosis. The patient was taken to the cardiac catheterization lab where he was found to have a proximal LAD 80-90% stenotic lesion (Figure 2).

**Figure 3:**
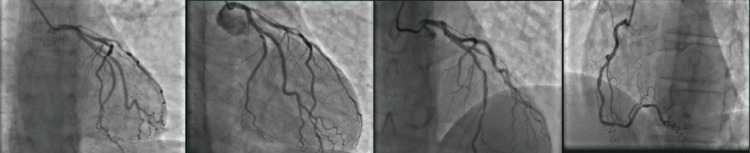
Coronary angiography views.

He was also found to have 70% stenosis of mid-LAD, 90-95% stenosis of the diagonal branch, and 50-60% stenosis of the ostial aspect of the first marginal artery (Figure 3).

Because of his critically severe ostial LAD disease that was not amenable for percutaneous coronary intervention, as well as a diagonal and circumflex marginal disease associated with acute ischemic cardiomyopathy, he was taken emergently to the operating room for definitive surgical revascularization.

## Discussion

Wellens' syndrome is an important clinical consideration for the management of patients presenting with chest pain, whether that be in the emergency room or the primary care office in the outpatient setting. These patients can have normal to elevated cardiac biomarkers and upon investigation with angiography, display critical narrowing of the LAD.

Wellens' syndrome was first described by de Zwaan et al. in 1982 in patients who were admitted for chest pain as a characteristic ECG pattern that correlated to patients with critical stenosis in the LAD [2]. Wellens' syndrome can be split into two subtypes. The more common Wellens type A subtype includes biphasic T waves; the less common Wellens type B subtype includes deeply and symmetrically inverted T waves [3]. This patient displays ECG findings suggestive of having both the common and less common type. In patients with a consistent ACS clinical presentation, Wellens' ECG patterns are correlated with severe stenosis of the LAD with 99% specificity for type A and 97% specificity for type B [1]. In de Zwaan’s initial study group, 75% of patients found to have these T wave patterns on ECG went on to have anterior wall myocardial infarction even when treated with medical therapy (de Zwaan). Because of the possibility of critical clinical ramifications, early detection of these patterns by medical professionals is essential.

With regards to the incidence of Wellens, Zhou et al. conducted a retrospective control study in a hospital in Beijing to study the incidence, risk factors, and long-term outcomes of Wellens' syndrome. A review of all ACS cases that presented to the hospital suggested that the overall incidence of Wellens was approximately 5.7% [4]. In terms of common presentations, Wellens was noted to present most commonly as a patient with a non-ST elevation myocardial infarction, though it has been described in prior studies as also commonly presenting with unstable angina. Furthermore, compared to non-Wellens ACS patients, cases of Wellens' syndrome often occur in patients with new onset cardiovascular disease, marking an important clinical distinction and supported by our particular patient’s presentation too.

Providers need to be aware of several things regarding the care of patients with suspected Wellens' syndrome. First and foremost, patients with suspected Wellens' syndrome should undergo cardiology consultation. Oftentimes, cardiac stress testing is avoided in this population as stressing the myocardium could precipitate a myocardial infarction or arrhythmia, and early coronary intervention is recommended as definitive treatment involves coronary stenting to the LAD or coronary artery bypass graft surgery [1]. Also, obtaining serial ECGs in a patient with chest pain is important as the usual ECG findings associated with Wellens' syndrome can be observed with a currently chest pain-free patient and a patient with high-grade LAD stenosis can be without the usual ECG findings associated with Wellens' syndrome [1].

## Conclusions

Wellens' syndrome is a type of ACS associated with critical stenosis of the left anterior descending coronary artery. Patients will present with chest pain and ECG findings including biphasic T waves or T wave inversions in leads V2 and V3. These specific ECG findings consistent with Wellens' syndrome are important for all physicians to recognize as they are associated with a serious cardiovascular condition and potentially poor outcomes if not identified. Specifically, if not treated promptly, the evolution can be an acute infarction with elevation of the ST segments. More emphasis should go into educating healthcare professionals about recognizing these critical ECG findings so that timely and appropriate interventions can be initiated for these patient populations.
